# Competence and Attitude of Family Physicians towards Sexuality Regarding Their Sexual Orientation, Age, or Having a Partner—Survey Study and Validation

**DOI:** 10.3390/ijerph191711029

**Published:** 2022-09-03

**Authors:** Javier Ramírez-Santos, Gracia Castro-Luna, Manuel Lucas-Matheu, Tesifón Parrón-Carreño, Bruno José Nievas-Soriano

**Affiliations:** 1 Almería Health District, Andalusian Health Service, 04008 Almería, Spain; 2 Nursing, Physiotherapy, and Medicine Department, University of Almería, 04120 Almería, Spain

**Keywords:** sexuality, primary care, family physicians, attitude, competences, procedures, questionnaire, validation

## Abstract

Background: The main objective of this study was to assess different aspects of family physicians (sex, age, sexual orientation, or having a partner) regarding their competencies, attitudes, and procedures towards their patients’ sexuality. We also sought to develop a valid questionnaire to perform this task. Methods: A cross-sectional study was performed among family and community medicine physicians in southeast Spain. Results: A total of 259 family physicians participated. Overall, 69.9% were women, 80.7% were heterosexual, 80.7% had a partner, and 50.6% had not received specific sexology training. Homosexual physicians showed a slightly more positive attitude toward sexuality. Training in sexuality established differences in competencies and procedures, but no differences were found in the attitude regarding whether the physicians had a partner or their training. While younger ages were correlated with a more positive attitude, the global score was positively correlated with the age of the professionals. Conclusions: Competences, attitudes, and knowledge of procedures do not depend on whether the professional has a partner, but there may be slight differences regarding attitude when considering the sexual orientation of the physicians. The attitude toward sexuality may not depend on previous training. Albeit younger family physicians have a more positive attitude, all providers become more involved with sexuality as they gain professional experience.

## 1. Introduction

The World Health Organization (WHO) recognizes sexual health as an essential aspect of the life of human beings and strives to promote recognition and attention to sexual rights through policies, education, and integration in health systems [1]. Despite this, the training of professionals around sexual health is scarce, and sexuality continues to be ignored in clinical practice [2,3]. Sexually transmitted infections affect about 365 million people per year, and it is a mandatory point to address in primary health care [4].

Family physicians have an essential role in this regard, being the first to be consulted on these issues and being able to detect problems in the sexual sphere of their patients [2,5,6]. Elements such as longitudinally in time allow an approach based on greater confidence [2,7,8]. In addition, they have patients in consultation who associate various types of pathologies, from diabetes to cardiovascular diseases, which affect the sexual sphere to a different extent, making difficulties in this matter more prevalent [9,10,11]. They generally have a good predisposition to attend to the sexuality of their patients, but there are several clear barriers to overcome [5]. Physicians’ attitudes are influenced by various factors, such as patients’ age, health status, or socioeconomic status, as well as their usual focus on family planning and sexually transmitted infections [5,6,11].

The level of training in sexual health among professionals is usually related to a more excellent approach to patients’ sexuality in their clinical practice, and understanding the development of this training is a priority to improve the skills of professionals [6,12]. The lack of training, consequently, conditions low confidence to address these issues, a poor perception of available therapeutic options and an underestimation of the prevalence of these difficulties, so they can be infra-diagnosed [12]. There is often an assumption that the sexual sphere passes into the background or that its deterioration is expected when other diseases appear [10]. The main barriers are that the patient and the doctor feel discomfort when dealing with these issues and time pressure. The latter is the most described by professionals [13,14,15,16].

Family doctors tend not to ask about sexuality and focus on the biological rather than psychological aspects of their patients’ sexuality [10,11,17]. However, despite the modesty of doctors, most patients do not feel invaded when asked about sexual aspects, even when the reason for consultation is another [11]. These barriers could be overcome by improving the training of professionals, something the professionals have demanded [12,14,16,18]. Regarding recording the sexual clinical history, again, the studies find that elements such as intimacy or lack of time play against [3,6,13,14,15]. In recent years, patients’ sexuality has become an enlightened area to address in their health, although they remain reluctant to consult about it. Evidence suggests that patients feel comfortable with physicians asking about their sexuality when it is appropriately approached, building stronger relationships with them. However, asking about sexuality exposes underlying concerns that patients would not consult otherwise [11].

There have been studies that present a nonstandardized method in their analyses [5], with a qualitative approach [10,11] or focused on patients [19]. However, no standardized questionnaires have been found to assess family physicians’ work regarding sexuality quantitatively. In addition, previous studies have focused on patients’ sexual orientation and physicians’ behavior towards it [20,21,22], but our research did not find studies in which doctors’ sexual orientation was considered.

Therefore, the main objective of this study was to assess the family physicians’ competence and attitude regarding sexuality quantitatively. To perform this, we assessed three spheres of family physicians’ practice regarding patients’ sexuality: their attitudes or mindset, their aptitudes or capacity to approach this issue, and their procedures or actual labor in this area. A questionnaire was developed to perform this task. We also sought to assess the validity of the questionnaire.

## 2. Materials and Methods

### 2.1. Design of the Study

A cross-sectional observational study was performed to evaluate physicians’ performance toward patients’ sexuality according to their attitudes, aptitudes, and procedures in clinical practice. The study was carried out among family and community medicine physicians in primary care of the Andalusian Health Service in Almería, located in southeastern Spain. This location was selected for reasons of plausibility and methodological soundness. According to the Spanish National Statistics Institute, these professionals attended to a population of 731,792 inhabitants as of 1 January 2022 [23]. The questionnaire was developed from scratch based on data obtained from a literature review and the author’s experience. The questionnaire collected five demographic aspects (sex, age, sexual orientation, if the participants had a partner, and sexology training); and fifty qualitative items that assessed the participants’ competence, attitude, and procedures. The questionnaire obeyed a Likert scale format, where participants responded one to four, where one meant «never» or «totally disagree» and four, «always» or «totally agree».

### 2.2. Sample Size Estimation

The sample size was calculated utilizing the Epi Info™ app, from Atlanta CDC, with the following parameters: population size of 731,792 [23], 80% confidence interval, and a level of precision of 5%. These parameters indicated a required sample size of 164 participants. The authors decided to collect at least 250 responses to lower potential auto selection bias and to comply with the classic rule established by Kline et al. [24] of using two to twenty subjects for each questionnaire item for the factorial analysis.

### 2.3. Eligible Population and Recruitment

The eligible populations were family and community medicine physicians in the Andalusian Health Service primary care in Almería. Population peculiarities were not contemplated, as the subject of the study were physicians and not their patients. The questionnaire was sent by email from the Almeria Primary Care Management of the Andalusian Health Service to the professionals potentially eligible to participate in the study. The inclusion criteria were: being family and community medicine physicians, working in the Andalusian Health Service in Almería, working in primary care, and being able to speak and read Spanish fluently. The exclusion criteria were: not meeting any of the referred inclusion criteria and not wishing to participate in the study, despite meeting the criteria.

### 2.4. Questionnaire Validation

Content validation was performed by an expert panel made up of nine family physicians and sexologists. The tool’s reliability was measured using Cronbach’s alpha [25]. The split-half method was used to assess the stability as the questionnaire could not be retested with the same users [26]. The adequacy of the exploratory factor analysis (EFA) was determined through the analysis of Bartlett’s test and the Kaiser–Meyer–Olkin (KMO) measure. For construct validity, the qualitative items of the questionnaire were evaluated through exploratory factor analysis. Confirmatory factor analysis (CFA) was conducted using AMOS software’s maximum likelihood estimation technique. This method read the structures determined in the exploratory factor analysis. The goodness of fit was assessed using the most typical fit indices employed in the literature [27]: the root mean square error of approximation (RMSEA), the normed fit index (NFI), the non-normed fit index (NNFI), or Tucker–Lewis index (TLI), and the comparative fit index (CFI).

### 2.5. Statistical Analyses and Review Board Approval

Statistical analyses and the exploratory factorial analysis were performed using SPSS version 28 (IBM Inc., Armonk, NY, USA). Univariant and bivariant analyses were conducted. The statistical software AMOS version 26.0.0 (IBM Inc., Armonk, NY, USA) was used for confirmatory factor analysis. The study was conducted in accordance with the Declaration of Helsinki. Informed consent was shown at the beginning of the questionnaire. Personal data were not collected. The confidentiality of the participants was absolute as no personal data were collected or stored, and the researchers only could access completely anonymous questionnaires. Although the responses were anonymous and, therefore, participants could not be identified, the questionnaires were stored in encrypted servers of the Andalusian Health Service. This study was approved by the Research and Ethics Committee of Nursing, Physiotherapy, and Medicine Department of the University of Almeria (Spain), with approval number EFM 205/2022.

## 3. Results

### 3.1. Sociodemographic Features

Two hundred and fifty-nine family and community medicine physicians participated in the research. Their mean age was 37.3 years (range 24–65), with a standard deviation (SD) of 11.8. One hundred eighty-one (69.9%) were women, two hundred and nine (80.7%) were heterosexual, two hundred and nine (80.7%) had a partner, and one hundred thirty-one (50.6%) had not received any specific training related to sexology (Table 1).

### 3.2. Validation of the Questionnaire

In the evaluation by the expert panel, the resulting content validity index was 1.00 for the questionnaire, on a scale of 0 to 1, with 1 being the best possible value. The Kaiser–Meyer–Olkin measure of sampling adequacy was 0.763, and Bartlett’s test for sphericity was 1654.4 with 66 degrees of freedom and a *p*-value < 0.001. The split-half method did not detect significant differences in the domains or the global evaluation (Table 2).

These results indicated the model’s suitability for exploratory factor analysis. This analysis was performed using principal component analysis and allowed to exclude 38 of the 50 initial items from the questionnaire. The analysis identified four domains that explained 77.8% of the variance (Table 3).

The first domain was defined by four items, the second and third domains by three items, and the fourth by another two items (Table 4).

These domains were interpreted by analyzing the items within each one (Table 5). The first domain defined professional procedures on sexuality, the second domain defined professional competence, the third domain defined professional attitude, and the fourth domain defined professional procedures on family planning.

The Cronbach’s alpha for the global questionnaire was 0.903 (Table 6). All the domains achieved Cronbach’s alpha values of 0.795 and above. The domain with the highest value was domain 2, while the domain with the lowest value was domain 4.

**Figure 1:**
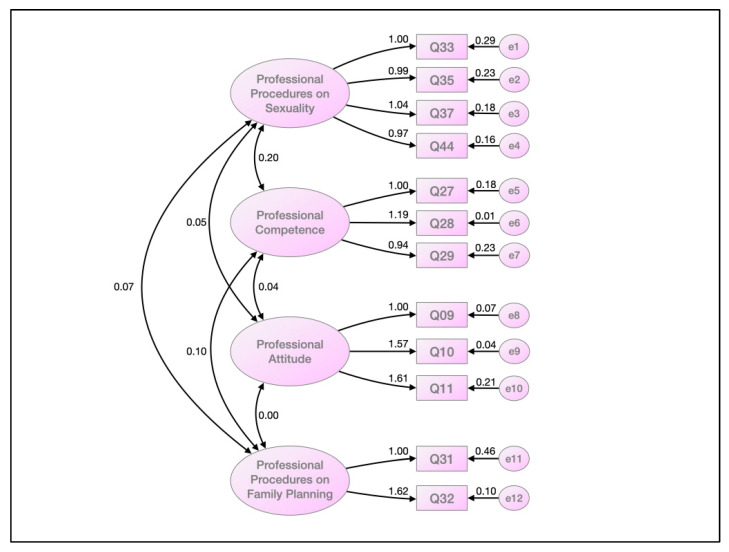
Measures of internal consistency of the construct by confirmatory factor analysis, applying maximum likelihood estimation method.

In the confirmatory factor analysis (Figure 1), by applying the maximum likelihood estimation method, the four constructs defined in the EFC were confirmed, with the items exhibiting correlations from 0.94 to 1.62. The highest correlation among the domains was 0.20, and the lowest was 0.00.

Regression values, critical ratio, standard errors, and significances are shown in (Table 7). Critical ratio weights were high, and the disparities were significant in all the parameters.

The model’s goodness of fit was estimated through the following indexes (Table 8): the NFI was 0.944, the NNFI (or TLI) value was 0.954, and the CFI value was 0.972. The value of the magnitude evaluation of the RMSEA was 0.061.

### 3.3. Univariant Analysis

Regarding calculating the domain scores based on the responses given by the study participants, the scores given by the participants were adjusted by the number of items of each domain and converted to a 10-point scale (Table 9). The domain that obtained the highest score was professional attitude, with a total of 9.5 points out of 10. The lowest scoring domain was professional procedures on sexuality, with 5 points. The overall score of the questionnaire was 7 points.

### 3.4. Bivariant Analysis

When analyzing the scores of the participant regarding their sex (Table 10), significant differences were found in domain two (professional competence), domain three (professional attitude) and the global score.

When analyzing the participants’ scores regarding their age (Table 11), there was a positive correlation between the global score and the age of the participants. Furthermore, the domain analysis showed statistically significant differences in three domains: in domains one and four, related to the professional procedures on sexuality and family planning, the correlation was positive, while in domain three, professional attitude, the correlation was negative. Thus, in this last domain, higher scores were given by younger participants.

The analysis of the scores of the participants regarding if they had a partner (Table 12) showed no statistically significant differences in any of the domains or the global score.

When analyzing the scores of the participant regarding their sexual orientation (Table 13), significant differences were found only in domain three, Professional Attitude, where heterosexual participants scored slightly lower than the other groups. Pairwise comparison showed that the heterosexual participants scored lower than the bisexual ones (*p* = 0.032; Kruskal–Wallis test).

The analysis of the participants’ scores regarding sexology training (Table 14) showed that the participants with sexology training scored higher in domains one, two, four, and the global score. These differences were statistically significant. However, no differences were found in the scores of the third domain, Professional Attitude, regarding this aspect.

## 4. Discussion

The main objective of this study was to assess the family physicians’ work regarding sexuality quantitatively through a questionnaire that evaluated their attitudes (that is, their disposition in this regard), their aptitudes (what they could do) and the procedures (what they currently did) of professionals of primary care regarding the sexuality of their patients. We also sought to assess the validity of the questionnaire to perform this specific task.

### 4.1. Sociodemographic Features

Most of the physicians that participated in this research were women, which can be representative of the current composition of primary care physicians, as stated by other authors [28]. This finding also agrees with the available data from the Andalusian Health Service [29]. The mean age of the doctors in our sample is similar to other similar research based on surveys performed on physicians [30]. An important aspect to consider is that there are studies assessing potential homophobia among doctors [31,32], but we have not found research that describes the percentage of heterosexual, homosexual, or bisexual doctors. Therefore, this research could be a pioneer in not only describing this aspect but also correlating it to the competencies, attitudes, and knowledge of procedures regarding sexology. Few studies have neither assessed having a partner regarding these aspects. Although some studies analyze the training in sexology regarding professional attitudes towards sexuality [33], they are scarce.

### 4.2. Validation of the Questionnaire

The exploratory factor analysis excluded 38 items from the initial 50-item questionnaire. This process allowed us to define a much shorter validated questionnaire, also easier to fulfill, based on the 12 items that contributed most to the construct of the questionnaire. This figure may appear significant but aligns with other studies in other ambits [34,35]. Moreover, the exclusion of those items permitted us to define better the four domains using fewer items. These domains were assessed based on the items which defined them, as stated by other authors [27]. Cronbach’s alpha coefficient is the most used method to evaluate the internal consistency of a questionnaire [27], and the value obtained can be regarded as excellent, according to other authors [27,34]. The split-half method, used in the same period or when other techniques such as test-retest cannot be used [36], verified the stability of the questionnaire.

The confirmatory factorial analysis was utilized to establish the questionnaire’s underlying conceptual structure [37]. Albeit the evaluation of RMSEA can be subjective, weights under 0.08 are considered indicative of a good fit, and in our case, it was much lower. The rest of the parameters used to evaluate the model’s goodness of fit were the most used in the literature [27], and all of them were close to one, indicating a near-to-perfect fit.

### 4.3. Univariant Analysis

Contrary to some authors who state that physicians’ attitudes toward sexology could be improved [38,39], the domain that defined their attitude toward sexology achieved the highest score among our physicians. However, as the overall score of the questionnaire was lower, and the score of the professional procedures on the sexuality domain achieved the lowest score, we agree with the affirmations of these same authors, who propose improving role modeling and education or multicomponent implementation programs to improve health professionals’ knowledge and competence when addressing sexuality issues with their patients [38,39].

### 4.4. Bivariant Analysis

The analysis of the different domains of the questionnaire regarding different aspects of the family physicians, such as sex, sexual orientation, training in sexuality, or if they had a partner, showed some interesting findings. Similar to other research [20,21], women scored higher in the domain that defined professional attitude. However, men scored higher in the domain that defined professional competence and the global score of the questionnaire, while other authors state that healthcare professionals have limited knowledge and confidence when addressing sexuality [21,39]. A plausible interpretation of this finding is that perhaps men may be more confident than women in their sexology skills and attitudes, even subjectively. Another important finding is that no differences were found in the scores of the domains or the global score regarding if the family physicians had a partner. This finding may be innovative, and it can help compare future research.

Some authors have described that heterosexual healthcare providers have a more positive attitude toward sexuality when they address heterosexual people [22]. However, in our research, we found two interesting findings: albeit heterosexual participants scored lower in the attitude domain, and pairwise comparison showed that the heterosexual participants scored lower than the bisexual ones, the *p*-value was slightly under the significance value. Even more, in the rest of the domains or the global score, no significant differences were found regarding the sexual orientation of the Family physicians. Therefore, we can conclude that the attitude toward sexuality was independent of the sexual orientation of the healthcare providers, excepting a possible worse attitude from heterosexual professionals. However, given the slight differences, this specific finding should be studied in more detail in future research.

It seems logical that the professionals with higher training in sexuality scored higher in the global score and the domains that defined the professional procedures on sexuality, family planning, and professional competence. However, contrary to several authors’ findings [6,12,40,41,42], we found no differences in the attitude of the professionals regarding their training in sexuality. We believe this is an exciting finding, as our interpretation is that if family physicians’ attitudes toward sexuality is the same, independently of their training in sexuality, it is because finally, professionals, whether trained in sexology or not, are at least beginning to be aware of the importance of adopting a proactive attitude to this issue.

The last aspect analyzed, the age of the family physicians, shows some striking results not reflected in the literature. It seems logical that younger physicians score higher in the domain that defines the attitude toward sexuality, while older participants score higher in the domains that define the knowledge of professional procedures on sexuality and family training. However, there was a positive correlation between the age of the physicians and the global score of the questionnaire. A possible conclusion of these findings is that, independently of their age, family physicians seem to become more involved with their patients’ sexuality as they gain professional experience. We believe that this is another critical finding that seems logical and promising.

A significant interpretation of our results is the potential applicability for clinical practice and professional training programs’ development. The validation of this questionnaire was intended to allow further application abroad, nationally or internationally, to comprehend family medicine physicians’ situation towards sexuality. This potential future research may allow the development of strategies to improve the situation when required. The data obtained from its appliance could also raise awareness among professionals about the relevance of education in these matters. Future research could dig into themes such as practitioners’ flaws and the resources they consider more helpful to acquiring sexuality concerns in their day-to-day work.

### 4.5. Limitations and Strengths

This research has some limitations. The most important is the selection bias due to various factors. Our sample was obtained from the family physicians of a province of 731,792 inhabitants, but it could not be representative of other regions or countries. In addition, participation was voluntary, which contributes to the potential selection bias. We must also consider that the questionnaire and the study were in Spanish. Larger sample sizes could also help increase the confidence interval. These aspects must be considered when assessing the external validity of our conclusions, which should be interpreted with caution.

This research also has some strengths. The most important one is that the questionnaire obtained excellent results in the exploratory and confirmatory factor analyses and the reliability studies. These aspects give almost complete validity to the final questionnaire and support the possibility of using it in future studies. Another important strength is that the study has been performed with current real healthcare professionals from primary care, and some of the aspects assessed are innovative. Thus, our findings can be helpful for current clinical practice and future research.

## 5. Conclusions

This study could be the first to describe the percentage of heterosexual, homosexual, and bisexual family physicians or the percentage with a partner and correlate these aspects to their competencies, attitudes, and knowledge of procedures regarding sexology and family planning in their patients. While female family physicians may show a more positive attitude toward sexuality, males feel more confident globally. Competences, attitudes, and knowledge of procedures are the same, independent of whether the professional has a partner, and there may be slight differences regarding attitude when considering the sexual orientation of the physicians. One of the most important findings is that the attitude toward sexuality does not depend on their previous training in this topic. Even more, albeit younger family physicians have a more positive attitude toward sexuality, all providers seem to become more involved with their patients’ sexuality as they gain professional experience. These are critical findings that can break some clichés. Finally, the developed questionnaire is a valid tool to assess these aspects and could be translated and culturally adapted to other languages or countries for future research.

## Figures and Tables

**Table 1:** Sociodemographic aspects.

Age	Mean	SD *
	37.3	11.8
Sex	n	%
Women	181	69.9
Men	78	30.1
Sexual orientation	n	%
Heterosexual	209	80.7
Bisexual	25	9.7
Homosexual	22	8.5
Other	3	1.2
Partner	n	%
Yes	209	80.7
No	50	19.3
Sexology training	n	%
None	131	50.6
Readings	91	35.1
Courses	33	12.7
Postgraduate training	4	1.5
Total	259	100.0

* SD = Standard Deviation.

**Table 2:** Split-half method.

Domain	Half	n	Mean	SD *	*p*-Value **
First	1st	130	8.1	2.6	0.424
	2nd	129	8.0	2.5	
Second	1st	130	7.7	2.1	0.780
	2nd	129	7.7	2.3	
Third	1st	130	11.2	1.3	0.444
	2nd	129	11.1	1.3	
Fourth	1st	130	6.5	1.4	0.520
	2nd	129	6.6	1.6	
Total	1st	130	33.5	5.1	0.574
	2nd	129	33.3	5.3	

* Standard deviation; ** Mann–Whitney U test.

**Table 3:** Total explained variance.

Component	Total	% of Variance	Accumulated %
1	4.590	38.2	38.3
2	1.977	16.5	54.7
3	1.465	12.2	66.9
4	1.300	10.8	77.8
5	0.494	4.1	
6	0.444	3.7	
7	0.402	3.4	
8	0.372	3.1	
9	0.325	2.7	
10	0.278	2.3	
11	0.253	2.1	
12	0.101	0.8	

Extraction method: principal component analysis.

**Table 4:** Rotated component matrix.

	Component			
	1	2	3	4
Q09			0.828	
Q10			0.881	
Q11			0.836	
Q27		0.899		
Q28		0.918		
Q29		0.789		
Q31				0.914
Q32				0.883
Q33	0.817			
Q35	0.790			
Q37	0.828			
Q44	0.810			

Extraction method: principal component analysis. Rotation method: Varimax with Kaiser normalization. Rotation converged in 5 iterations.

**Table 5:** Domains detected.

Domain 1. Professional Procedures on Sexuality.
Q33. During your medical history, do you ask about your patients’ sexual health?
Q35. I am interested in the sexual satisfaction of my patients.
Q37. I talk about sexuality not only at the request of my patients.
Q44. I spend time in my practice addressing the sexuality of my patients.
Domain 2. Professional competence.
Q27. I can counsel my young patients on ways to improve their sexuality.
Q28. I can counsel my middle-aged patients on ways to improve their sexuality.
Q29. I can counsel my elderly patients on ways to improve their sexuality.
Domain 3. Professional Attitude.
Q09. Talking about sexuality with young patients is essential.
Q10. Talking about sexuality with middle-aged patients is essential.
Q11. Talking about sexuality with elderly patients is essential.
Domain 4. Professional Procedures on Family Planning.
Q31. I am aware of the protocol for the voluntary interruption of pregnancy.
Q32. I can provide advice on family planning.

**Table 6:** Cronbach’s Alpha of the questionnaire and its domains.

Domain	Cronbach’s Alpha
Professional Procedures on Sexuality	0.868
Professional Competence	0.902
Professional Attitude	0.797
Professional Procedures on Family Planning	0.795
Global Questionnaire	0.903

**Table 7:** Confirmatory factor analysis. Regression Weights, standard errors, critical ratios, and significances.

Regression Weights		Estimate	Standard Error	Critical Ratio	*p*
Q33	Domain 1	1.000			
Q35	Domain 1	0.989	0.083	11.862	***
Q37	Domain 1	1.041	0.083	12.555	***
Q44	Domain 1	0.970	0.077	12.534	***
Q27	Domain 2	1.000			
Q28	Domain 2	1.191	0.058	20.421	***
Q29	Domain 2	0.941	0.059	15.837	***
Q09	Domain 3	1.000			
Q10	Domain 3	1.569	0.140	11.245	***
Q11	Domain 3	1.610	0.152	10.614	***
Q31	Domain 4	1.000			
Q32	Domain 4	1.619	0.433	3.743	***

*** significant.

**Table 8:** Confirmatory factor analysis. Model adjustment measures.

Adjustment Measure	Default Mode
NFI	0.944
RFI	0.910
IFI	0.972
TLI	0.954
CFI	0.972
RMSEA	0.061
LO 90	0.042
HI 90	0.079

**Table 9:** Scores of the Domains and Global Score.

Domain	n	Lowest	Highest	Mean	SD	Items	Adjusted Score *
Professional Procedures on Sexuality	259	4	16	8.1	2.5	4	5.0
Professional Competence	259	3	12	7.7	2.2	3	6.5
Professional Attitude	259	6	12	11.1	1.3	3	9.3
Professional Procedures on Family Planning	259	2	8	6.5	1.5	2	8.3
Global Score	259	19	48	33.4	5.2	12	7.0

* Adjusted by the number of items in each domain, on a 10-point scale.

**Table 10:** Scores regarding the sex of the participants.

Domain	Sex	n	Mean	SD	Items	Adjusted Score *	*p*-Value **
Professional Procedures on Sexuality	Men	78	8.5	2.5	4	5.3	0.083
	Women	181	7.9	2.5		5.0	
Professional Competence	Men	78	8.3	2.1	3	7.0	0.002
	Women	181	7.4	2.2		6.3	
Professional Attitude	Men	78	10.9	1.4	3	9.0	0.040
	Women	181	11.2	1.3		9.3	
Professional Procedures on Family Planning	Men	78	6.6	1.5	2	8.3	0.689
	Women	181	6.5	1.5		8.3	
Global Score	Men	78	34.3	5.4	12	7.3	0.039
	Women	181	33.0	5.1		7.0	

* Adjusted by the number of items in each domain, on a 10-point scale; ** Mann–Whitney U Test.

**Table 11:** Scores regarding the age of the participants.

Domain	Correlation Coefficient	*p*-Value *
Professional Procedures on Sexuality	0.213	0.001
Professional Competence	0.065	0.294
Professional Attitude	−0.131	0.035
Professional Procedures on Family Planning	0.475	<0.001
Global Score	0.242	<0.001

* Rho de Spearman.

**Table 12:** Scores regarding if the participants had a partner.

Domain	Partner	n	Mean	SD	Items	Adjusted Score *	*p*-Value **
Professional Procedures on Sexuality	Yes	209	8.2	2.6	4	5.1	0.054
	No	50	7.4	2.2		4.6	
Professional Competence	Yes	209	7.7	2.2	3	6.4	0.983
	No	50	7.7	1.9		6.4	
Professional Attitude	Yes	209	11.2	1.2	3	9.3	0.508
	No	50	10.9	1.5		9.1	
Professional Procedures on Family Planning	Yes	209	6.6	1.4	2	8.3	0.062
	No	50	6.1	1.7		7.6	
Global Score	Yes	209	33.7	5.2	12	7.0	0.202
	No	50	32.2	5.1		6.7	

* Adjusted by the number of items in each domain, on a 10-point scale; ** Mann–Whitney U Test.

**Table 13:** Scores regarding the sexual orientation of the participants.

		n	Mean	SD	Items	Adjusted Score *	*p*-Value **
Professional Procedures on Sexuality	Heterosexual	209	8.0	2.6	4	5.0	0.388
	Homosexual	22	8.7	2.5		5.5	
	Bisexual	25	8.2	2.3		5.2	
	Other	3	9.3	2.3		5.8	
Professional Competence	Heterosexual	209	7.6	2.1	3	6.4	0.620
	Homosexual	22	8.2	2.1		6.8	
	Bisexual	25	7.9	2.5		6.6	
	Other	3	8.0	3.5		6.7	
Professional Attitude	Heterosexual	209	11.0	1.4	3	9.2	0.043
	Homosexual	22	11.5	0.7		9.6	
	Bisexual	25	11.6	0.9		9.6	
	Other	3	12.0	0.0		10.0	
Professional Procedures on Family Planning	Heterosexual	209	6.6	1.5	2	8.2	0.178
	Homosexual	22	6.2	1.7		7.7	
	Bisexual	25	6.4	1.3		8.1	
	Other	3	5.3	0.6		6.7	
Global Score	Heterosexual	209	33.2	5.3	12	6.9	0.524
	Homosexual	22	34.6	4.8		7.2	
	Bisexual	25	34.2	4.5		7.1	
	Other	3	34.7	5.5		7.2	

* Adjusted by the number of items in each domain, on a 10-point scale; ** Kruskal–Wallis Test.

**Table 14:** Scores regarding sexology training of the participants.

		n	Mean	SD	Items	Adjusted Score *	*p*-Value **
Professional Procedures on Sexuality	None	131	7.4	7.4	4	4.6	0.001
	Readings	91	8.3	8.3		5.2	
	Courses	33	9.7	9.7		6.0	
	Post-graduate	4	12.3	12.3		7.7	
Professional Competence	None	131	7.2	7.2	3	6.0	0.001
	Readings	91	7.8	7.8		6.5	
	Courses	33	9.2	9.2		7.6	
	Post-graduate	4	9.3	9.3		7.7	
Professional Attitude	None	131	11.2	11.2	3	9.3	0.732
	Readings	91	11.0	11.0		9.2	
	Courses	33	11.1	11.1		9.3	
	Post-graduate	4	11.0	11.0		9.2	
Professional Procedures on Family Planning	None	131	6.3	6.3	2	7.9	0.015
	Readings	91	6.7	6.7		8.4	
	Courses	33	7.0	7.0		8.8	
	Post-graduate	4	7.3	7.3		9.1	
Global Score	None	131	32.1	32.1	12	6.7	0.001
	Readings	91	33.8	33.8		7.0	
	Courses	33	36.9	36.9		7.7	
	Post-graduate	4	39.8	39.8		8.3	

* Adjusted by the number of items in each domain, on a 10-point scale; ** Kruskal–Wallis Test.

## Data Availability

Not applicable.
